# Hidden gems: Scattered knowledge hampered freshwater jellyfish research over the past one‐and‐a‐half centuries

**DOI:** 10.1002/ece3.70350

**Published:** 2024-09-29

**Authors:** Florian Lüskow, Nicholas Bezio, Luciano Caputo, Xupeng Chi, Henri J. Dumont, Krishan D. Karunarathne, Pablo J. López‐González, Maciej K. Mańko, Guillaume Marchessaux, Kentaro S. Suzuki, Evgeny A. Pakhomov

**Affiliations:** ^1^ Department of Earth, Ocean and Atmospheric Sciences University of British Columbia Vancouver British Columbia Canada; ^2^ Institute for the Oceans and Fisheries, University of British Columbia Vancouver British Columbia Canada; ^3^ Department of Biology University of Maryland Baltimore Maryland USA; ^4^ Facultad de Ciencias, Instituto de Ciencias Marinas y Limnolóogicas Universidad Austral de Chile Valdivia Chile; ^5^ Centro Transdisciplinario de Estudios Ambientales y Desarrollo Humano Sostenible Universidad Austral de Chile Valdivia Chile; ^6^ CAS Key Laboratory of Marine Ecology and Environmental Sciences Institute of Oceanology, Chinese Academy of Sciences Qingdao China; ^7^ Laboratory of Marine Ecology and Environmental Sciences Qingdao National Laboratory for Marine Science and Technology Qingdao China; ^8^ Department of Biology Gent University Ghent Belgium; ^9^ Department of Ecology Jinan University Guangzhou China; ^10^ Department of Aquaculture and Fisheries Wayamba University of Sri Lanka Makandura Sri Lanka; ^11^ Departamento de Zoología, Biodiversidad y Ecología Acuática Universidad de Sevilla Sevilla Spain; ^12^ Laboratory of Plankton Biology, Department of Marine Biology and Biotechnology University of Gdańsk Gdynia Poland; ^13^ Laboratory of Ecology, Department of Earth and Marine Science University of Palermo Palermo Italy; ^14^ Sustainable System Research Laboratory Central Research Institute of Electric Power Industry Abiko Japan

**Keywords:** Cnidaria, *Craspedacusta*, Hydrozoa, interdisciplinary research, limnology

## Abstract

Freshwater jellyfish (= limnic medusa‐budding hydrozoans, FWJ) are a small group of cnidarians found on all continents except Antarctica in temperate to tropical latitudes. Members of this group belong primarily to three genera: *Astrohydra*, *Craspedacusta*, and *Limnocnida*. While *Astrohydra* and *Limnocnida* are typically restricted to the islands of Japan, Africa, and the Indian subcontinent, one species or potential species complex, *Craspedacusta sowerbii*, became globally invasive. Despite research going back about one‐and‐a‐half centuries, little is known about their phylogeny and ecology compared to marine jellyfish. Recent species distribution modelling, however, showed that by 2050, *C. sowerbii* will potentially extend their distribution ranges due to global warming to high‐latitude ecosystems and be present (medusa stage) for an extended time in the seasonal limnic production cycle. An increase in their relative ecological importance with temporal and spatial spreading is hypothesised. Only recently, it has been shown that the trophic roles of polyps and medusae and their prey overlap with other ecosystem members. In addition, medusa behaviour may cause trophic cascades and alter vertical nutrient distributions. However, polyps and other benthic life cycle stages are understudied. In globally, changing freshwater ecosystems that may become more accommodating for FWJ, an improved understanding of their population biology and ecosystem ecology is urgently needed. In this integrative review, we, therefore, explore reasons for the hampered historical research progress, contrast developments with those of marine cnidarians, compile and publish alongside an extensive and unprecedented literature database, and formulate avenues for future directions in FWJ research.

## Graphical abstract

Approximately 150 years of research on freshwater jellyfish globally have resulted in a considerable amount of information. However, this is not comprehensively available to most researchers worldwide. Scattered information allows only for a fragmented view of the research field, which mediated by climate change received increasing importance. Trends, patterns, recent developments and urgent research needs are critically reviewed.

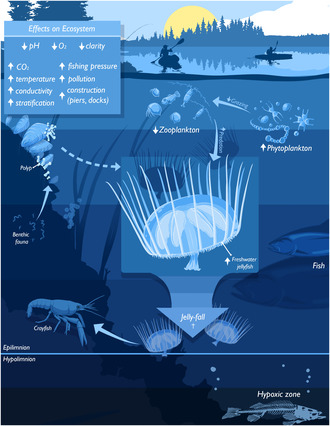

## 1 GELATINOUS ORGANISMS IN OCEANS AND LAKES AND PRESENT‐DAY KNOWLEDGE GAPS

Gelatinous zooplankton (GZ) include diverse taxonomic and functional groups (e.g., cnidarians, ctenophores, thaliaceans; see Haddock (2004) for marine fauna and Dumont (2007) for freshwater fauna) but have several traits in common. These include translucent tissues/bodies, high water contents facilitating near neutral buoyancy, fast growth, generally large size of adults, and often a lack of hard structures making them fragile and often difficult to sample (Larson, 1986; Madin & Harbison, 2001). Gelatinous fauna had been studied for over a century, with efforts focussed on coastal and near‐surface oceans and significantly fewer studies conducted in the deep sea (Boero et al., 2008; Haddock, 2004; Pugh, 1989). Gelatinous zooplankton are sensitive to environmental change and respond quickly to, for instance, minor temperature increases. In marine ecosystems, the effects of global warming have been associated with an increase in the frequency of GZ blooms, which in turn, has relevant adverse consequences for local and regional biodiversity (Boero et al., 2016). Consequences have also been linked to negative effects on human enterprises (reviewed by Purcell, 2012). However, gelatinous fauna is pivotal for the functioning of food webs and biogeochemical cycles (Brodeur et al., 2019; Luo et al., 2022; Tinta et al., 2021). Marine and freshwater ecosystems vary in many aspects and one of them is the presence of gelatinous fauna. Gelatinous zooplankton in aquatic (= freshwater) ecosystems are primarily composed of rotifers (gelatinous sheath). Neither the phylum Ctenophora Eschscholtz, 1829 nor the subphylum Tunicata Lamarck, 1816 evolutionarily managed to establish species in limnic environments. Species in the phylum Cnidaria Hatschek, 1888, particularly members of the class Hydrozoa Owen, 1843, are an exception (Dumont, 2007).

Limnic medusa‐forming hydrozoans (= freshwater jellyfish, FWJ) are a species‐poor group (potentially less than 40 species), distributed primarily in tropical to temperate latitudes in both hemispheres (Dumont, 1994; Jankowski et al., 2008). Besides FWJ, limnic systems host a variety of other non‐medusa‐budding hydrozoans like *Hydra* Linnaeus, 1758. Freshwater jellyfish primarily consist of members of the three genera *Astrohydra* Hashimoto, 1981, *Craspedacusta* Lankester, 1880a, 1880b, and *Limnocnida* Günther, 1893 (Jankowski & Anokhin, 2019, Figure 1). In addition, there are several less well‐studied genera such as *Australomedusa* Russell, 1970, *Keralica* Khatri, 1984, and *Mansariella* Malhotra, Duda & Jyoti, 1976 are either doubtful in taxonomic validity, regionally confined, or inhabiting inland salt lakes (e.g., Bayly, 1971; Jyoti & Sehgal, 1984; Khatri, 1984). Jellyfish in the latter genera are, for the purpose of this review, left aside and will be discussed elsewhere. Freshwater jellyfish share a metagenetic life cycle, in which asexually reproducing benthic polyps and sexually reproducing pelagic medusae alternate (Bouillon, 1957; Folino‐Rorem et al., 2016; Marchessaux & Bejean, 2020). Other benthic stages are frustules and podocysts. The various life cycle stages are studied with considerably different efforts across species.

**FIGURE 1:**
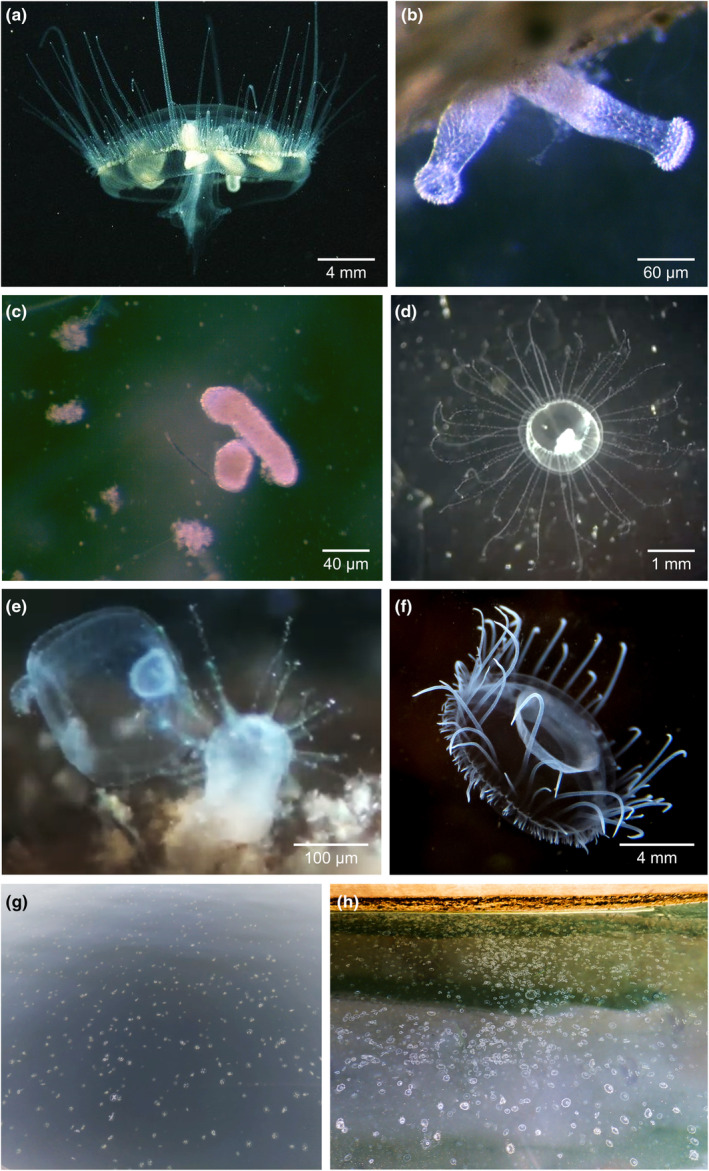
Morphologies of three freshwater jellyfish species in different life cycle stages. (a) Medusa of *Craspedacusta sowerbii*, (b) Polyp colony of *C. sowerbii*, (c) Frustules of *C. sowerbii*, (d) Medusa of *Astrohydra japonica*, (e) Polyp of *A. japonica* in the process of budding a medusa, and (f) Medusa of *Limnocnida indica*. Seasonal mass occurrences (blooms) of (g) *C. sowerbii* and (h) *L. tanganjicae* are common around the world, and in Africa and on the Indian subcontinent, respectively. Picture credits: (a) C. Dehondt from DORIS (http://doris.ffessm.fr), (b,c) G. Marchessaux, (d,e) Still frames from video clips made by K. Saotome, (f) J. W. Barton, (g) A. Brown at Killarney Lake, British Columbia, and (h) A. P. h. Bose at Lake Tanganyika, Zambia. Scale bars differ among pictures.

Historically, the field of FWJ research is rather young. By coincidence, *Craspedacusta* medusae were first observed in and described from a water lily tank in Regent's Park, London, UK, in 1880 (Lankester, 1880a, 1880b). Prior to this, only one doubtful report of a freshwater medusa exists from the Netherlands in 1762 (Hummelinck, 1938). In 1893, the medusae of *Limnocnida* were first described from Lake Tanganyika (Günther, 1893) and in 1912 from the Indian subcontinent (Annandale, 1912). Only recently, in 1981, the polyps and later medusae of *Astrohydra* (currently a monospecific genus) were discovered in Japan (Hashimoto, 1981, 1985). Currently, it is unclear how *Calpasoma dactylophorum* Fuhrmann, 1939, a species known from outside Japan (e.g., Fuhrmann, 1939; Matthews, 1966; Peterson et al., 2022) with similar polyp phenotypes relates to *Astrohydra*. Evidence of an earlier detection from Lewis et al. (2012) exists. However, history did not start there. In ancient Chinese, there are four dialects or local names of FWJ: 士淑水母: The pronunciation of this name is similar to ‘dead‐water jellyfish’ in the Hangzhou dialect where it was found; 马鼻子–Horse nose or horse nasal mucus; 桃花扇–Peach Blossom Fan; 桃花鱼–Peach Blossom Fish. The last one, ‘Peach Blossom Fish’ is the most common name. It is unknown who first coined these names. Old notes mention these FWJ were found at the time of peach bloom, and the jellyfish look like falling petals of the flowers. Kimura (1937) reviewed ancient Chinese literature–some dating back to the Song dynasty in the year 1250. Despite centuries‐old written proof of existence, FWJ were kept aside as an ‘abnormality’ by many GZ researchers and limnologists. Occurrences were simply noted in hundreds of reports published since 1880. Despite some noteworthy reviews on FWJ ecology (e.g., Acker & Muscat, 1976; DeVries, 1992) and their global phylogeography (Dumont, 1994; Jankowski et al., 2008; Jankowski & Anokhin, 2019; Mayer, 1910; Oualid et al., 2019), a wholistic one‐and‐a‐half centuries‐spanning meta‐analysis of the globally available literature is lacking, potentially because of the high publication language diversity and inaccessibility of many literature pieces. However, regional‐ and global‐scale climate change, as well as alterations of species distribution ranges, urge for this challenging wholistic meta‐analysis.

The FWJ *Craspedacusta sowerbii* Lankester, 1880a, 1880b is one of the most widespread aquatic invasive species. However, its global distribution remains uncertain due to ephemeral appearances of the medusae and a general lack of information in various aquatic environments. *Craspedacusta sowerbii* is native to the Yangtze River basin in China but has been introduced to various parts of the world through natural and human‐mediated activities as documented in various studies in North America, Europe, Asia, South America, and Australia (Dumont, 1994). The species is present in various freshwater systems, such as lakes, reservoirs, ponds, and slow‐moving rivers (Dumont, 1994; Marchessaux et al., 2021). Despite the occasional and short‐lived presence of the medusae in water bodies, *C. sowerbii* and non‐medusa‐budding freshwater hydrozoans have been extensively studied for about one‐and‐a‐half centuries. The expansion of this species can be attributed to various factors, with human activities, e.g., the transportation of aquatic plants or movement of watercrafts, facilitating its spread to new freshwater ecoregions. Additionally, the species' ability to form dormant stages called podocysts allows for surviving adverse environmental conditions and subsequently favouring secondary dispersal via zoochory through the newly colonised areas (Acker & Muscat, 1976).

**FIGURE 2:**
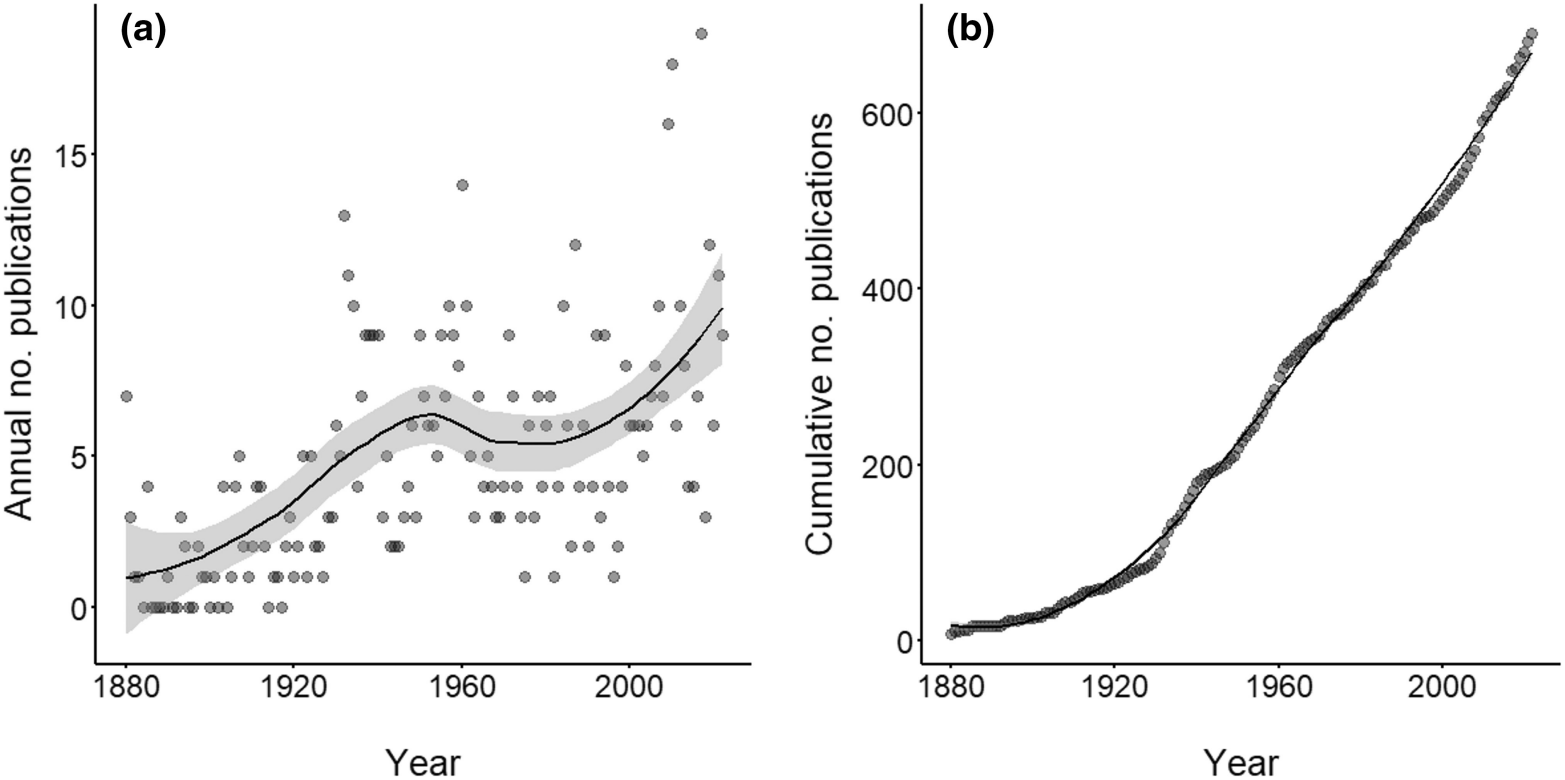
The (a) Annual and (b) Cumulative numbers of primary papers on freshwater jellyfish worldwide (*N* = 690) between 1880 and 2022. Papers on several FWJ genera were counted twice or thrice. Smoothed regression functions indicate the temporal nonlinear trend. A generalised linear model (Poisson error structure, log‐link) shows a significant positive trend (GLM, *z* = 11.2, *p* < .001) of annual publication numbers over time.

Environmental factors such as temperature, water quality and transparency, and prey availability also determine its expansion (Caputo et al., 2018). Temperature, in particular, is the environmental factor influencing the distribution of *C. sowerbii* the most (Marchessaux et al., 2022; McClary, 1959). Hence, climate change is expected to contribute significantly to the future expansion of this species (and others) globally. Predictions suggest that this species will invade high‐latitude regions in both hemispheres over the next 80 years, forcing ecological consequences upon already threatened freshwater ecosystems (Marchessaux et al., 2021). Hydrozoans (medusae and polyps) are increasingly recognised as important components of freshwater ecosystems (e.g., Gießler et al., 2023), maybe not only because of increasing bloom frequency (Marchessaux et al., 2021, 2022) but also because of the scientific literature becomes more comprehensive and accessible for the global researcher community (Figure 2). Due to the difficulties to access diverse publications principally published in local languages and the increase in interest on these FWJ, a review was performed to create an extensive and accessible database.

The primary objectives of this review were to (i) explore the numerical succession of scientific publications on FWJ since 1880 (first species description) concerning studied genera, life cycle stages, used languages, and chosen journals, to pinpoint key reasons for hampered scientific advancement. While freshwater and inland hydrozoan diversity is larger than covered in this integrative review, the three chosen genera (*Astrohydra*, *Craspedacusta*, and *Limnocnida*) constitute the vast majority–systematically, geographically, and likely ecologically. We then set out to (ii) explain the succession of FWJ research using historical and geopolitical developments. The hampered knowledge development on FWJ was then (iii) comparatively illustrated by showing the number of papers on *Craspedacusta* as the best‐researched FWJ genus in contrast to those of the well‐studied freshwater hydrozoan *Hydra* and marine hydromedusa *Aequorea* Péron & Lesueur, 1810. It was further our goal to (iv) generate a comprehensive literature database (https://doi.org/10.1594/PANGAEA.962186) that will allow future investigators to include century‐old studies and works published not only in English. Finally, we (v) synthesised information on trends in FWJ research and formulated avenues for future research directions.

## 2 FRESHWATER JELLYFISH LITERATURE DATABASE

At first, a literature search on freshwater jellyfish (FWJ) in the Web of Science (WoS) and Google Scholar was performed using all kinds of relevant search keywords such as ‘*Craspedacusta*’, ‘Freshwater Jellyfish’, ‘*Limnocnida*’, and ‘*Astrohydra*’. Many, already known, papers were not listed using this approach. We thus started to extend the literature search ‘by hand’, i.e., manually following citation pathways back to their sources, to cover the time between 1880 (first species description) and September 2023. Works were included in the database when meeting the following selection criteria:

- peer‐reviewed research papers and notes, reviews, and book chapters.
- no theses and dissertations.
- no symposium and conference contributions.
- no newspaper articles.
- grey literature, i.e., articles in smaller regional journals, was chosen on a case‐to‐case base, and
- scientific work in all languages was considered.

Although we can assume that most literature was included, some articles (likely early studies) may have been missed. The database contains information in the following categories, which will make future searches for specific topics considerably easier: Genus, short reference, paper title, publication year, publication decade, journal, volume, issue, language, life cycle stage, and the continent of the first author's primary affiliation (https://doi.org/10.1594/PANGAEA.962186). In total, the FWJ database contains 697 entries (partly counted multiple times because several FWJ genera were addressed), covering the three major FWJ genera *Astrohydra*, *Craspedacusta*, and *Limnocnida*. Literature that addresses several genera is included twice or, in a few cases, thrice. Entries belonged to more than 400 journals (or books). Three life cycle stages were indicated, i.e., frustule, medusa, and polyp. Literature on planulae and podocysts was negligible. We differentiated between papers published in Chinese, Dutch, English, French, German, Japanese, and Russian and clustered publications in other less‐often used languages as ‘Other’ (thirteen languages contributing less than 10%). Institute affiliations of FWJ researchers were located on all continents except Antarctica. For clustering, Russia and Turkey were part of Europe, and whereas Mexico was part of North America, Central American countries were grouped into South America. The generated database complements previously created synthesis efforts by Lüskow et al. (2021) [http://www.int‐res.com/articles/suppl/b030p069_supp2.xlsx] and Marchessaux et al. (2021) [https://doi.org/10.1594/PANGAEA.936074] that focussed on environmental conditions at medusa bloom times and the global distribution and associated key environmental parameters of medusa occurrence, respectively.

A generalised linear model (GLM) was used to explore the significant increase in the number of publications per decade over time (Poisson error structure, log‐link) in the R package “lme4” (Bates et al., 2015). We were interested in testing whether commonly believed stereotypes of FWJ research are true, i.e., the medusa stage is more often studied compared with benthic life cycle stages, *Craspedacusta* is more frequently studied than other genera, English is the primary language used to communicate FWJ research, and most FWJ research is conducted at European universities and research institutes. To test these hypotheses, we applied *t*‐tests (*α* level of .05) to the number of research papers per decade in the various pairwise groupings, e.g., papers on the medusae stage versus frustules and podocysts (15 decades = 15 data points per group, balanced design). To meet the assumption of normality of data and homoscedasticity of variances, data were log_10_‐transformed. For investigating publication language and continent affiliation of primary investigators over decades, non‐metric multidimensional scaling (NMDS) analysis was performed. All statistical analyses and visualisations were performed in R (R Core Team, 2023) version 4.2.2 using the package “ggplot2” (Wickham, 2016) and QGIS 3.10.10 “A Coruña”.

Since the first description of FWJ (*C. sowerbii*) in 1880, research has progressed, with a global gross average of five papers per year. Despite large interannual variability, there is an upward trend of annual publication numbers (as a proxy for knowledge gain) over time that peaked in 2017 with 19 literature pieces (Figure 2). While the initial phase of FWJ research until the 1940s indicated exponential knowledge gain, the annual number of publications has since then become near‐linear totalling in 2022 with 690 papers. The lack of exponential growth of annual publication numbers indicates a small, continuously productive researcher community and the lack of an extraordinary event attracting outside interest, e.g., biomedical or biotechnological, to the research field. The research progress on FWJ is compared with that of a marine jellyfish and a non‐medusa budding freshwater hydrozoan species below.

## 3 FACTORS CONTRIBUTING TO HAMPERED PROGRESS IN FRESHWATER JELLYFISH RESEARCH

### 3.1 Misspelling and changed species names

Revision and relocation of species, genera, or entire families are common in taxonomy. However, this practice is especially widespread in the small hydrozoan family Olindiidae Haeckel, 1879 (order Limnomedusae Kramp, 1938). Not only did the genera *Craspedacusta* and *Limnocnida* move between orders Trachymedusae Haeckel, 1866 and Limnomedusae several times but also species names often changed. For instance, *C. sowerbii*, the best‐studied FWJ species, was redescribed by various authors more than half a dozen times since its formal description by Edwin Ray Lankester in 1880 (Table 1, WoRMS, 2023). Eventually, all names (partly originating from a separate description of polyp and medusa) are considered synonyms, and the species is currently accepted under its original name *C. sowerbii*.

**TABLE 1:** Historic development of species name, synonyms, and variants of *Craspedacusta sowerbii* from its initial description and in the following half a century.

Paper/authority	Species name	Comment
Lankester (1880a)	*Craspedacusta sowerbii*	
Lankester (1880b)	*Limnocnidum sowerbii*	= *C. sowerbii*
Allman (1880)	*Limnocnidum victoria*	*= C. sowerbii*
Potts (1885)	*Microhydra ryderi*	*= C. sowerbii*
Oka (1907)	*Limnocnidum kawaii*	*= C. sowerbii*
Mayer (1910)	*Craspedacusta kawaii*	*= C. sowerbii*
Payne (1924)	*Craspedacusta ryderi*	*= C. sowerbii*
Roch (1924)	*Microhydra germanica*	*= C. sowerbii*
Gaw and Kung (1939)	*Craspedacusta sowerbyi* var. *kiatingi*	*= C. kiatingi*

*Note*: Timeline is informed, in parts, by the World Register of Marine Species (WoRMS, accessed on 27 May 2023).

Similarly, the nominal species *L. tanganjicae* was in its original description spelt differently (*L. tanganyicae* Günther, 1893, WoRMS, 2023). A few years before, R. Böhm cited in von Martens (1883) reported the presence of this species in Lake Tanganyika and proposed the specific epithet ‘Tanganjicae’ for it, however, without providing a genus name. This was, thus, not a valid introduction of the species' name. A decade later, Günther (1893) proposed a new genus and validly described the species, crediting Böhm for the name. These nomenclatural changes, which happened for the most part about a century ago, continue to have effects on today's literature and searchability and generate the impression of uncertain taxonomic entities. Redescription of several genetic lineages, currently pooled in the *C. sowerbii* species complex, may change the nomenclature once again, in search of the linage that must retain the name *C. sowerbii*, and to decide what to do with other lineages and proposed names (either considered as separate species, subspecies, or simply synonyms) (Lüskow et al., 2021, 2023; Morpurgo et al., 2021; Oualid et al., 2019).

### 3.2 Underappreciation of life cycle stages other than medusae

**FIGURE 3:**
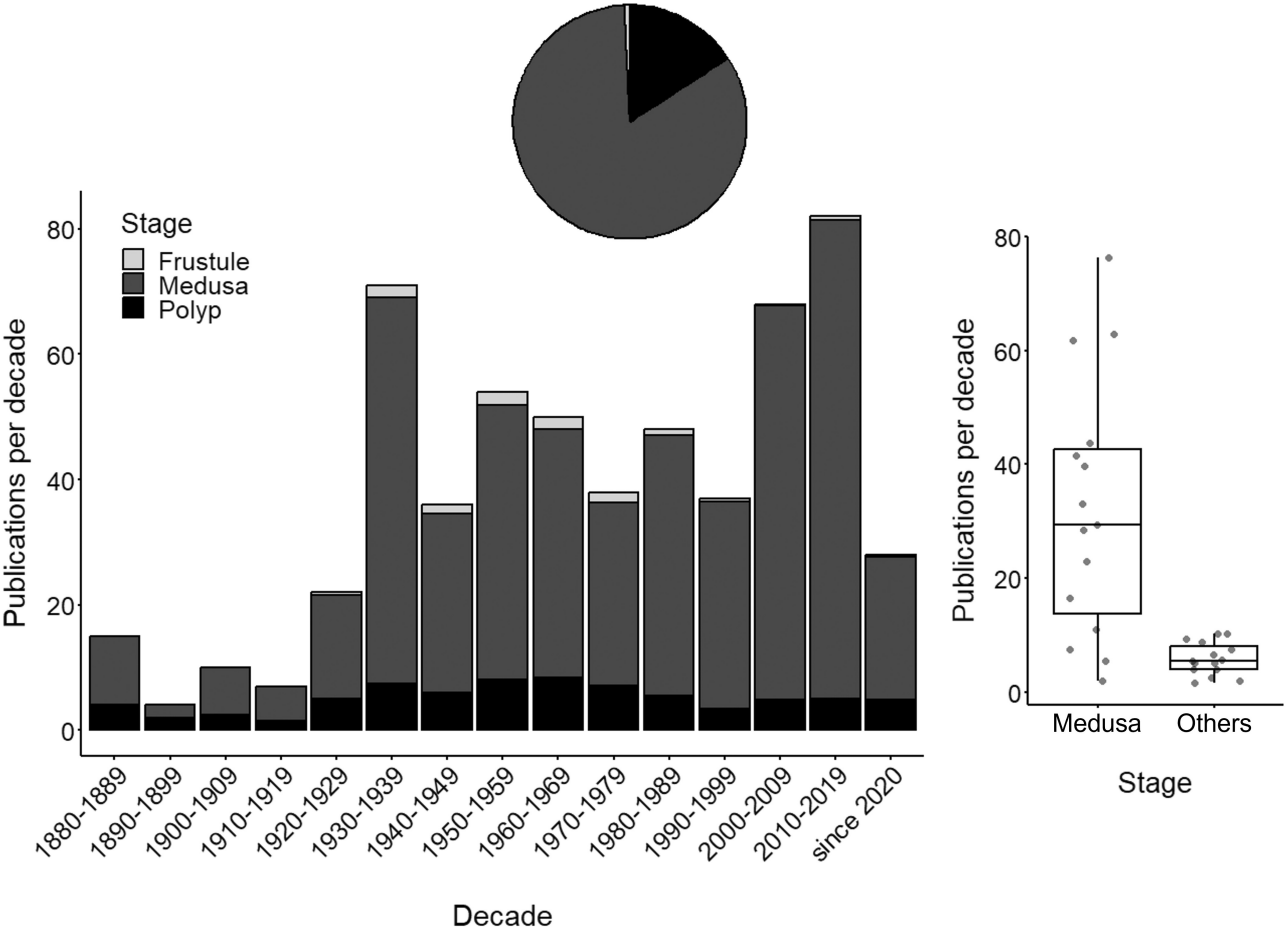
The decadal number of primary papers on *Craspedacusta* distribution, biology, ecology, and systematics worldwide (*N* = 570) from 1880 to 2023. The life cycle stage in focus (i.e., frustule, medusa, or polyp) is indicated. The number of publications per decade of the predominantly investigated life cycle stage (= medusa) is contrasted in a boxplot with the summed number of publications per decade of other life cycle stages. Line: Median; box: Interquartile range (IQR); whiskers: Max./min. value ≤1.5 × IQR above/below box.

The pelagic life cycle stage of FWJ, i.e., medusae, occurs only for a few weeks, maybe up to 3 months per year, whereas frustules, polyps, and other benthic life cycle stages have a cryptic lifestyle and remain hidden from most observers. The genus *Craspedacusta* is investigated as a best‐studied example. Caused primarily by their more conspicuous morphology and ecology, but also by the higher potential for health impacts on humans, medusae are significantly more often studied (>80%) than all other life cycle stages combined (Figure 3, *t*‐test with log_10_‐transformed data, *t*_1,28_ = 20.2, *p* < .001). It is noteworthy that the majority (81%) of studies covered one life cycle stage only (commonly the medusa), whereas only a few (about 16%) included two or all three (frustule, medusa, and polyp; about 2%) stages. Over the past one‐and‐a‐half centuries, only a dozen studies addressed planula larvae (https://doi.org/10.1594/PANGAEA.962186, primarily caused by the absence in unisexual populations) and podocysts, respectively. While research on benthic FWJ polyps was published since the species descriptions (Figure 3), research on the pelagic stage has governed our perception.

### 3.3 Predominance of research on *Craspedacusta*

**FIGURE 4:**
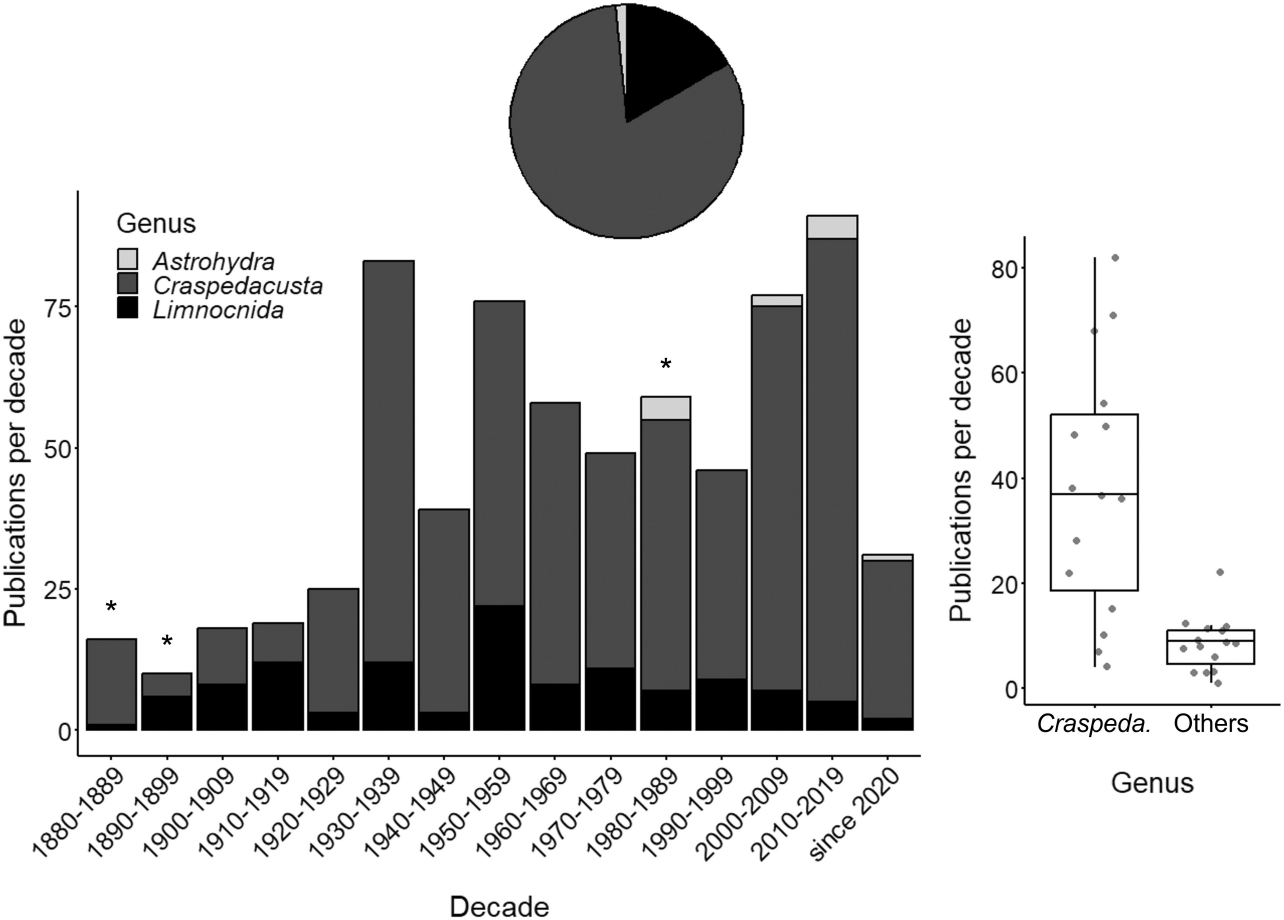
The decadal number of primary papers on *Astrohydra*, *Craspedacusta*, and *Limnocnida* distribution, biology, ecology, and systematics worldwide (*N* = 697) from 1880 to 2023. The genus in focus is indicated. The number of publications per decade of the predominantly investigated genus (= *Craspedacusta*) is contrasted in a boxplot with the summed number of publications per decade of other genera. Asterisks indicate the description decades of three key genera: *Astrohydra* Hashimoto, 1981, *Craspedacusta* Lankester, 1880, and *Limnocnida* Günther, 1893. Line: Median; box: Interquartile range (IQR); whiskers: Max./min. value ≤1.5 × IQR above/below box.

Research cannot be conducted without bureaucratic and monetary constraints. Thus, it is not surprising that scientists have (given the opportunity) studied local ecosystems and communities more intensively. As much of the global human population is situated in temperate latitudes (primarily in the northern hemisphere), it is not surprising that FWJ in these regions are investigated in more detail. This is especially true for genera with invasive species. Furthermore, various geopolitical situations, the extent of war zones, and other objectively dangerous world regions have historically and in present days hampered FWJ research. Currently, only members of the genus *Craspedacusta* are known to occur outside their native ranges (Marchessaux et al., 2021, 2022). Whether this is entirely true or may change due to climate change is up for debate. There are significantly more studies published on the genus *Craspedacusta* than on other genera combined (Figure 4, *t*‐test with log_10_‐transformed data, *t*_1,28_ = 21.3, *p* < .001). While in the first four decades since the description of *C. sowerbii* in London, UK, the proportion of *Limnocnida* studies was considerable and sometimes even exceeded 50% of the annual research output, *Craspedacusta* was in the 20th and 21st centuries the primarily studied FWJ genus. *Astrohydra* (first described in 1981) is currently regionally restricted to the Japanese islands and has not attracted much attention (Figure 4). It may be speculated that this is because of its minute size and limited potential to form blooms. In essence, our present perception of FWJ is primarily governed by *Craspedacusta*.

### 3.4 Diversity of publication languages

**FIGURE 5:**
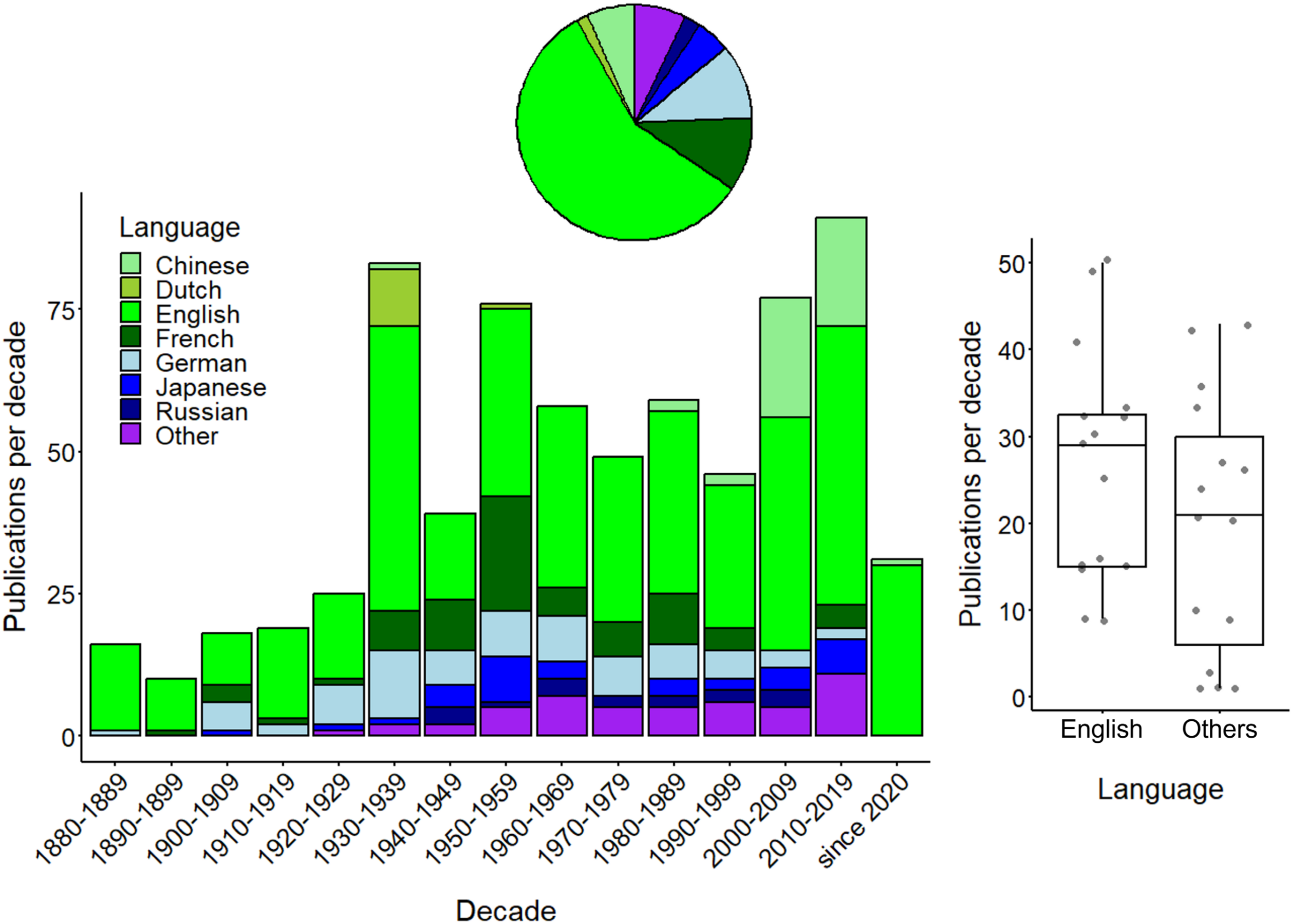
The decadal number of primary papers on freshwater jellyfish worldwide (*N* = 697) from 1880 to 2023. The number of publications per decade of the predominantly used language (= English) is contrasted in a boxplot with the summed number of publications per decade of other languages. Line: Median; box: Interquartile range (IQR); whiskers: Max./min. value ≤1.5 × IQR above/below box.

The publication language is tightly bound to research funding institutions, expected audiences, and habits that have changed throughout history. English, French, and German (and to a smaller degree Japanese) were the early publication languages (Figure 5). This strongly diversified in the first half of the 20th century. The research was now published in many languages, including Dutch, Portuguese, Russian, Spanish, and Ukrainian. After the dawn of the 21st century, most FWJ papers were published in Chinese and English (Figure 5). The colonial perspective of language use in FWJ research is addressed below. These three phases can also be seen in non‐metric multidimensional scaling space (NMDS using Bray‐Curtis measurements based on family densities, Figures S1–S3).

English, as the primary language used in international science, is also the most important language in communicating FWJ research. More than 50% of all papers were written in English. However, 43% (= 297 literature pieces) were in a language other than English, with German, French, and Chinese (in descending order) contributing the most. A comparison between the number of English and all non‐English literature published per decade revealed no significant difference (Figure 5, *t*‐test, *t*_1,28_ = 1.7, *p* = .196). While English literature is the most widely available and comprehensive source of information, much exists beyond. Multilingualism and internationalisation may be especially important in often regional‐scale FWJ research (discussed below). In other words, researchers who only read English FWJ literature miss about half of all available knowledge collated over one‐and‐a‐half centuries.

### 3.5 Colonial, economic crisis, and global wars signatures of FWJ research

**FIGURE 6:**
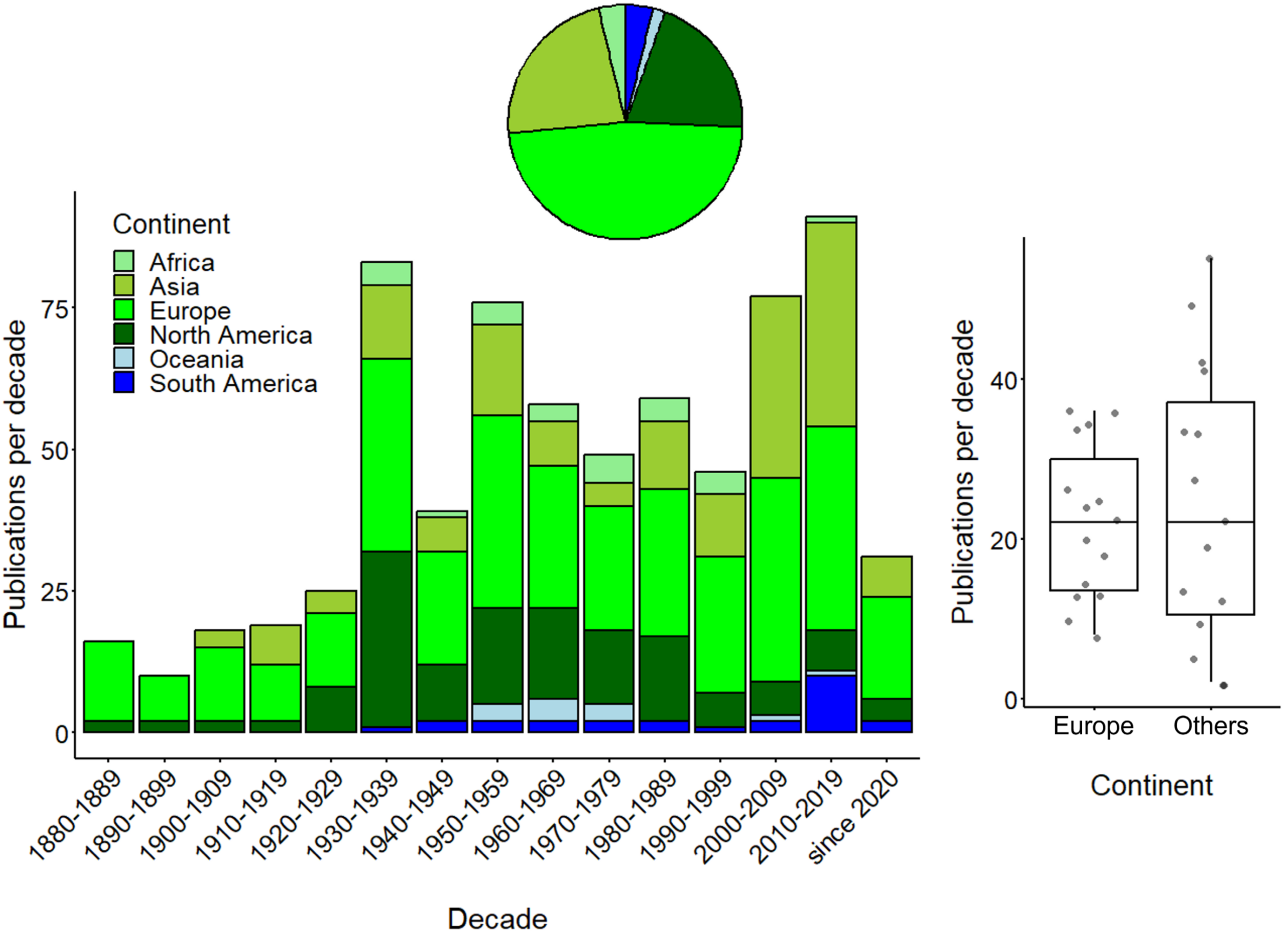
The decadal number of primary papers on freshwater jellyfish worldwide (*N* = 697) from 1880 to 2023. The first author's primary institutional (continent) affiliation is indicated. The number of publications per decade sorted by the primary institutional affiliation of the first author (= Europe) is contrasted in a boxplot with the summed number of publications per decade from other continents. Line: Median; box: Interquartile range (IQR); whiskers: Max./min. value ≤1.5 × IQR above/below box.

In line with language usage trends seen in the previous section, the early decades of FWJ research (1880s–1920s) were dominated by European and North American (to a smaller degree Asian) scientists (Figure 6). This is also apparent in the NMDS plot (Figure S2). Scientists are hereafter referred to as, for instance, European not by ethnicity, but by their primary affiliation to a research institution and continent. Naturalists and researchers from these continents had sufficient funds to undertake long‐distance travels, conduct in situ observations and early experiments, and publish their findings in accredited journals that can still be accessed today. The colonialism of European nations in Africa, Asia, and South America allowed natural explorers to travel abroad and investigate ecosystems so far unknown to the Western World. Despite an extensive global search, we could not find any literature that goes back before the description of *C. sowerbii* in the Royal Botanical Garden in London in 1880 (except a doubtful report by Hummelinck, 1938). Exceptions are the ancient Chinese notes mentioned in an early section of this review.

Only in the 1930s, did African and South American scientists publish their first works, and it should take another two decades for New Zealand and Australian scientists to do the same (Figure 6). Notably, the number of publications tripled in the 1930s, which at first glance surprises. The Great Depression that began in 1929 and ended the Roaring Twenties possibly caused a backsliding from expensive long‐distance travelling and research and allocation to more regional ecological communities. This regionalised research resulted in a boom of FWJ papers from all continents (except Oceania). As a result of Second World War‐related activities, e.g., military service, forced migration, and destruction of laboratories and collections, the numerical research output in the 1940s decreased by half. More specifically, the annual number of literature pieces dropped from 9 between 1937 and 1940 to 2–5 between 1941 and 1947 (Figure 2). As seen in many Western economies, research expenses (and so FWJ knowledge gain) doubled in the 1950s promoted by fear of and interest in the Manhattan Project and nuclear threat. Since then, the decadal number of papers has remained high but variable (between 45 and 85 papers). The considerable increase in literature pieces from Asian authors (primarily written in Chinese, Figures 5 and 6) after 2000 is again noteworthy. As argued above, Asian researchers focussed predominantly on regional communities, including FWJ, which is inexpensive compared to long‐distance travel. As documented by the increase in the number of Chinese papers, this research may address a more regional audience.

One aspect that should not be underappreciated is the connection between military and science. While marine researchers often piggyback on military vessels (coast guard or Navy) in many countries, this mutually fruitful collaboration is much less developed in river and lake settings (or missing entirely) as these are most often smaller water bodies and located inside national boundaries, not requiring military presence. Running research platforms such as vessels with well‐equipped laboratories is one of the major components of budgeting field‐based research projects. If this expense can be waived (or significantly reduced), research becomes considerably more feasible (marine research). The lack of such suggests the opposite (freshwater research).

**FIGURE 7:**
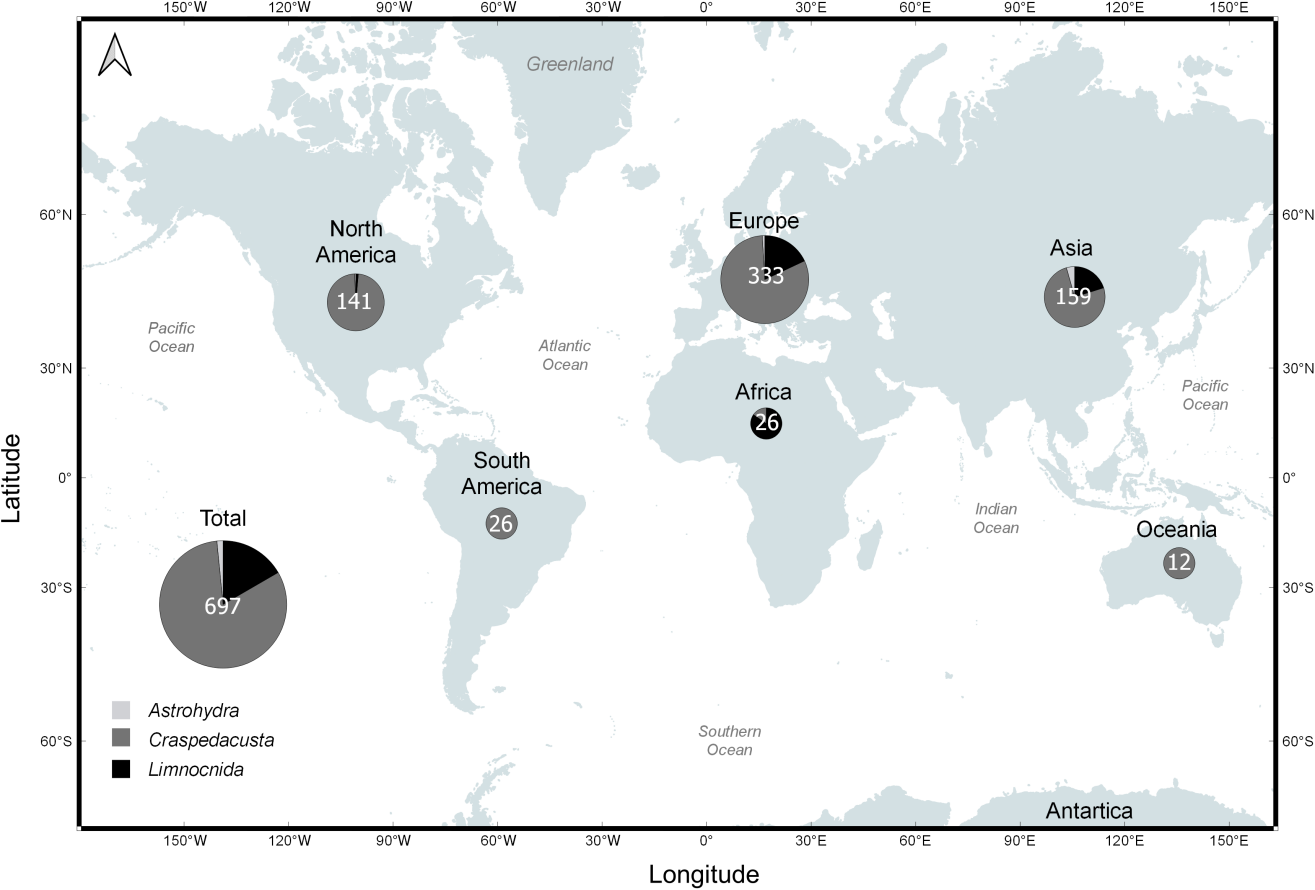
The cumulative research effort (indicated by the number of papers published on the three freshwater jellyfish genera [*Astrohydra*, *Craspedacusta*, *Limnocnida*]) from 1880 to 2023 split by continent of primary affiliation of the first author (*N* = 697).

This mixture of first author's affiliations used as a proxy for regional affinity with a dominance of Asian, European, and North American scientists seen from the 1930s onwards continues until today. A visualisation of continent affiliation of researchers in NMDS space showed that composition data are indistinguishable with the most remarkable clustering of early (1880s–1920s) decades with exclusively Asian, European, and North American affiliations. While the search for FWJ in the early decades was primarily oriented to foreign places, recent research has focussed more on ecosystems and communities near research institutes. The lack of such institutions with sufficient personnel and monetary support again explains why so little progress is made in Africa and Oceania. About half of all publications were written by scientists with European affiliations (Figures 6 and 7). However, as this pattern dissolved in the first half of the 20th century (partly linked to the end of colonialism in many places), European works are not significantly more abundant than all others combined (*t*‐test, *t*_1,28_ = 0.2, *p* = .690). Most of the FWJ research conducted in Europe (>80%) focussed on one genus (*Craspedacusta*). Similar values (or even 100% in Oceania and South America) contrast sharply with African efforts with >80% of papers dedicated to *Limnocnida* (Figure 7).

In summary, while most research today and historically is conducted by Asian, European, and North American scientists, investigations are made everywhere. We encourage scientists, especially from Africa, Oceania, and South America, to push FWJ research and find international synergies. In the sections below, we elaborate on, in our eyes, the most pressing directions of future works.

### 3.6 Scattered publications of FWJ research and missing theme‐lead journals

Peer‐reviewed articles on FWJ were primarily published in five journals: Science (32 papers), Hydrobiologia (21 papers), Nature (21 papers), Transactions of the American Microscopical Society (14 papers), and Zoologischer Anzeiger (14 papers). For details, the reader is referred to the PANGAEA database (https://doi.org/10.1594/PANGAEA.962186). While Science and Nature were primary research outlets in the second half of the 19th century and first half of the 20th century, in more recent decades, theme‐lead journals typically publishing FWJ research are lacking. Of the more than 600 listed FWJ papers, only about 15% occurred in the top‐ranked journals, i.e., peer‐reviewed journals with at least ten publications between 1880 and 2023. Approximately 85% of the papers were published in local or smaller journals or journals not particularly known for FWJ research, creating the impression of little progress in the research field. This is in sharp contrast to research on marine jellyfish, which relies heavily (but not exclusively) on some key journals, e.g., Journal of Plankton Research, Marine Biology, and Marine Ecology Progress Series. Most often, journals publish only one or two articles on FWJ (https://doi.org/10.1594/PANGAEA.962186).

## 4 HISTORIC SUCCESSION OF RESEARCH EFFORTS ON SELECTED FRESHWATER AND MARINE HYDROZOANS

When the number of primary publications on the best‐studied FWJ genus, *Craspedacusta*, is compared with that of *Aequorea*, a well‐studied marine hydromedusa, and *Hydra*, the best‐studied freshwater hydrozoan, vastly different trends are seen (Figure S3). To enable such comparison, the WoS was searched between 1880 and 2022 as ‘Year Published’ and with the genus name as ‘Topic’ (accessed on 2 May 2023). While the number of peer‐reviewed scientific papers on *Hydra* (an easy‐to‐cultivate model organism in developmental biology and genomics, whereas knowledge of its ecology, evolution, and systematics is relatively poor) grew exponentially since the 1960s, totalling 5673 papers in 2022 and approached since the 1990s a near‐linear increase, publication records on *Aequorea* built up, especially since the 1990s facilitated by the increasing usage of the green fluorescent protein (GFP) discovered in *Aequorea* with annual, on average, 31 new studies (*N* = 903). The historical development of publications on *Craspedacusta* is in sharp contrast to them. While the annual increment never exceeded seven papers according to WoS, in 2022, 124 on the FWJ genus are available (Figure S3). An analysis of covariance indicated significant effects of publication year (*F*_1,1_ = 3196.4, *p* < .001) and genus (*F*_1,2_ = 573.6, *p* < .001), as well as their interaction (*F*_1,2_ = 48.9, *p* < .001) on the log(x + 1)‐transformed number of publications.

*Craspedacusta*, as likely other FWJ genera, is vastly underrepresented in this, and potentially other, search engines. The reason for this is likely that many (smaller and not indexed) journals (often publishing in languages other than English) are not included in the search, which leads the investigator to believe in the apparent scarcity of knowledge. Our time‐consuming ‘by‐hand search’ showed, on the contrary, that FWJ research is surprisingly rich, and delivered about four times higher output than suggested by WoS. The comprehensive literature database published in PANGAEA (https://doi.org/10.1594/PANGAEA.962186) along with this study will enable future researchers to search more efficiently for relevant previous work and thus significantly contributes to pushing the field of FWJ research forward.

## 5 AWAKENING OF A NEW ERA – FRESHWATER JELLYFISH IN THE ANTHROPOCENE

Challenges freshwater ecosystems face that may favour (invasive) FWJ, are e.g., brownification, acidification, warming, and diversity loss (Caputo et al., 2018). *Craspedacusta sowerbii* is predicted to massively expand its global distribution until the end of the 21st century (Marchessaux et al., 2021) and to occur as medusae for longer periods in the seasonal cycle (Marchessaux et al., 2022). Whether this trend is also seen for species in the genera *Astrohydra* and *Limnocnida* is currently entirely unknown, but limited observations suggest they are more geographically restricted. Cross‐boundary collaborations are key to advancement in FWJ research; in contrast to research on marine jellyfish (often conducted outside national waters), FWJ most often occur inside national boundaries, and thus, research often lacks international character.

## 6 UNDERSTANDING TROPHIC ROLES

**FIGURE 8:**
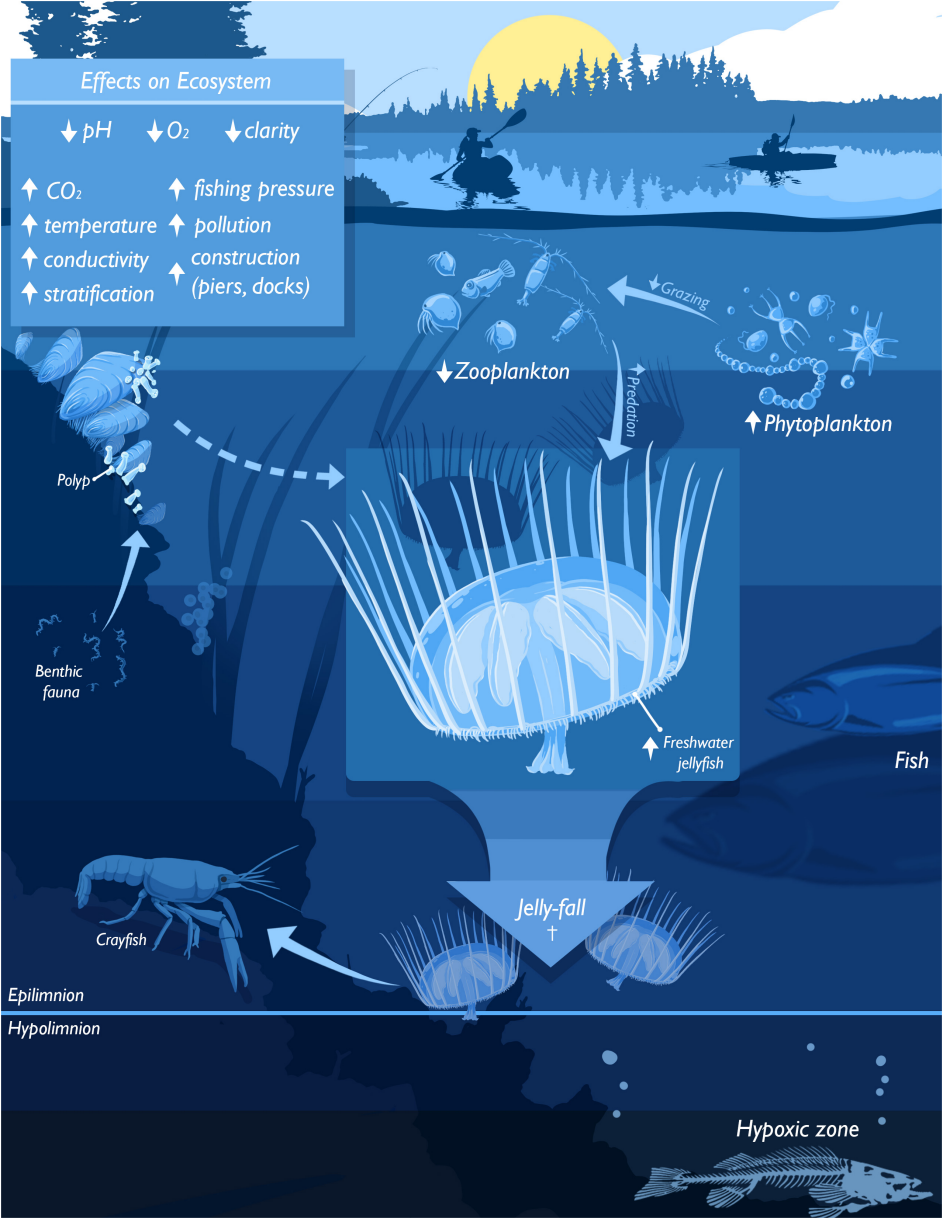
Major ecosystem environmental stressors and roles of freshwater jellyfish (FWJ) in the Anthropocene. Note that FWJ includes medusae, polyps, and other benthic life cycle stages. This scheme is mostly representative of temperate lake ecosystems with a *Craspedacusta* population, however, could be similarly shown for tropical or subtropical systems. In the box, climate change stressors, e.g., warming, increasing conductivity, CO_2_, stratification, and risk of bottom‐water hypoxia and decreasing dissolved oxygen and pH, are listed with mostly beneficial consequences for FWJ populations (see details in the text). Arrows in the main scheme indicate on the one side, medusa budding of polyps and on the other side, experimentally shown trophic interactions between benthic and pelagic functional groups. Trophic cascades are explained in the text. Schematic illustration not to scale.

Trophic structure, such as predator–prey and parasitic relationships, is a fundamental characteristic of ecosystems (Lafferty et al., 2008; Link, 2002). Since FWJ show broad distribution with considerable potential to expand even more under future climate change (Marchessaux et al., 2021, 2022), their trophic roles are important features to understand (Caputo et al., 2021; Figure 8). The polyp (and possibly other benthic stages) is the key stage for the invasion success of FWJ because it can have high longevity, and its asexual reproduction is a dominant way to maintain and increase populations. Besides habitat suitability, prey availability is one of the major determinants for polyp invasion success in new environments (Folino‐Rorem et al., 2016). The medusae are produced infrequently and appear over short periods. However, medusa biomass can be notably high during the bloom; thus, the medusae can strongly impact the ecosystem (Jankowski et al., 2005).

Both the polyp and medusa stages can be top predators in freshwater food webs and prey upon various zooplankton, such as copepods, cladocerans, rotifers, insect larvae, and even fish larvae (e.g., Dendy, 1978; DeVries, 1992; Gießler et al., 2023). The degree of FWJ predation on eggs, however, is uncertain. The trophic niches of polyps of FWJ and native *Hydra* species may overlap. Thus, the possibility of food competition and competitive exclusion has been discussed (Folino‐Rorem et al., 2016). A recent study using stable isotopes analysis, however, revealed that the polyps of *C. sowerbii* and *Hydra* occupied different trophic niches (Gießler et al., 2023): polyps of *C. sowerbii* tended to prey on benthic grazers, such as the cladoceran *Pleuroxus truncatus* (O. F. Müller, 1785), while *Hydra* polyps predated mostly zooplankton. The morphological difference between the two polyp species, where *Hydra* polyps have a more elongated body and tentacles in contrast to *C. sowerbii* polyps, might cause these different trophic niches. On the other side, the medusae prey mainly on zooplankton with a size of <5 mm. Several studies on the predatory impacts of *C. sowerbii* bloom have demonstrated that the food preferences of jellyfish are taxa such as cladocerans, copepods, and rotifers (e.g., Dodson & Cooper, 1983; Smith & Alexander Jr., 2008; Gießler et al., 2023; Figure 8), and thus they can compete for food with other species, like larvae of insects, e.g., *Chaoborus* Lichtenstein, 1800, and fishes (Gießler et al., 2023). During their bloom, predation of medusae has impacts not only on the number of prey organisms (negative impact) but also on phytoplankton biomass via trophic cascades resulting in increasing turbidity (positive effect, Jankowski et al., 2005), or even possibly on the vertical nutrient fluxes (Schachtl et al., 2023) and fish behaviour (Bose et al., 2019). Apart from the predatory effect, *C. sowerbii* kills, but does not eat organisms up to circa 9 mm long (Dodson & Cooper, 1983). While the medusa bloom can strongly impact the ecosystem, the sporadic and short‐term appearance of the medusae should enable their co‐occurrence with planktivores. Parasitism is widespread like in marine cnidarians, but little studied for FWJ. The parasitic amoeba *Hydramoeba hydroxena* Entz, 1912 (Rice, 1960) and protist *Trichodina pediculus* Ehrenberg, 1838 (Jarms, 1978) were found on medusae of *C. sowerbii* and have the potential to degrade the medusa quickly. This ciliate is typically a parasite of hydroids and fishes, may occur on amphibian tadpoles (Kazubski, 1991), and has also been treated as commensal on the tentacles of *C. sowerbii* specimens collected in the Thames River (Green, 1998). Ciliates of the order Mobilida Kahl, 1933 have been associated with severe disease and mortalities of freshwater fishes, causing high economic losses (van As and Basson 1986; van As & Viljoen, 1984), especially on cultured larval fish (García‐Magaña et al., 2019). Similarly, van As and Basson (1986) reported on *Trichodina* infestation of *L. tanganjicae* medusae in the Zambezi system. However, the frequency of such infections yet needs to be elucidated. In this sense, and depending on the duration and dispersion of medusae in the water bodies, FWJ can be considered an additional opportunistic host of or vector for these parasitic ciliates. Therefore, including FWJ species in regular monitoring programmes is strongly recommended.

Regarding the holistic trophic roles of FWJ, there remain considerable gaps in knowledge (Caputo et al., 2021; Figure 8), especially about the quantitative estimation of their diet, predators, and the ecosystem‐wide impacts of FWJ. As mentioned above, their feeding habits have been extensively investigated. However, only a single study (Gießler et al., 2023) quantitatively analysed the contribution of various prey groups to their assimilation using stable isotopes. However, such information is crucial to understanding the energy flow in ecosystems. In addition, microzooplankton can be underappreciated as prey items of FWJ due to their small body size. Small planktonic ciliates contribute considerably to the diet of the marine scyphomedusa *Aurelia coerulea* von Lendenfeld, 1884 (Kamiyama, 2011, 2023). Planktonic ciliates are usually abundant where FWJ occur. Thus, ciliates may be important prey for FWJ (Caputo et al., 2021).

The predators of FWJ, which are important to pinpoint for explaining their population dynamics and energy flow in ecosystems, are totally understudied (e.g., Lüskow et al., 2021). Only a few qualitative observations of crayfish and ducks feeding on medusae are known (Dodson & Cooper, 1983). Recently, Huyghe et al. (2023) identified *L. tanganjicae* medusae as prey of several clupeid fish species using stomach content metabarcoding. Medusae are nutritionally sufficient food for fish (at least juveniles) and of similar dry weight‐based energetic value as co‐occurring cladocerans, copepods, and rotifers (Lüskow et al., 2023). Marine cnidarian jellyfish had been seen as ‘trophic dead ends’ with only a few predators. The nutritional value, e.g., fatty acid composition, of marine gelatinous zooplankton is a rising concern for ecologists (Stenvers et al., 2020), lending counterevidence to the trophic dead end hypothesis, though is entirely unstudied in FWJ. New approaches, such as animal‐borne cameras and stomach content and faeces metabarcoding, have revealed that a wide range of marine predators consume them, including fishes, birds, turtles, and various invertebrates (Hays et al., 2018). Applying such approaches to FWJ will be vital to enhancing our knowledge about their trophic roles. The ecosystem‐wide impacts of FWJ have also been studied less. Microcosm/mesocosm experiments and ecological modelling are generally applied to understand the ecosystem‐wide effects of aquatic organisms. While there has been a small number of microcosm/mesocosm experiments (e.g., Jankowski et al., 2005; Salonen et al., 2012; Schachtl et al., 2023; Smith & Alexander Jr., 2008), no modelling studies exist at the time of article publication.

## 7 INCLUSION IN FOOD WEB, BIOENERGETIC, AND BIOGEOCHEMICAL MODELS

Climate change, eutrophication and/or brownification, deoxygenation, and biological invasions represent temperate lakes' most far‐reaching threats to biodiversity and ecosystem integrity (Jane et al., 2021; Sala et al., 2000; Wang et al., 2021). There is a consensus that global warming reinforces the eutrophication of already eutrophic lakes (Moss et al., 2011), which experience noticeable changes in food web structure and community composition (Jeppesen et al., 2000, 2020). Recent studies suggest that climate change, eutrophication, and declining water clarity have altered the physical and chemical environment of lakes, affecting biogeochemical cycling at local, regional, and even global scales (Meyer‐Jacob et al., 2019; Sepulveda‐Jauregui et al., 2018) and facilitating in many cases biological invasions of low‐solar radiation‐tolerant species (Caputo et al., 2018; Marchessaux et al., 2022). They also indicate that global change promotes increases in the productivity of aquatic ecosystems (Meerhoff et al., 2022) and that eutrophication (Beaulieu et al., 2019), particularly of temperate lakes with an enlarged duration of thermal stratification (Winder & Schindler, 2004), show a greater emission of greenhouse gases, such as methane and carbon dioxide.

The effects of eutrophication together with global warming have been associated with an increase in the frequency of blooms of invasive ctenophores and pelagic cnidarians, with adverse consequences for local and regional biodiversity (Brotz et al., 2012; Duarte et al., 2013; Purcell, 2012), public health and coastal tourism (Graham et al., 2014; Pimentel et al., 2005). Among the invasive freshwater cnidarians, only *C. sowerbii* is reported to have colonised both natural and artificial lake systems in various climatic regions, except Antarctica (Dumont, 1994). According to NASA and NOAA, recent years have been the warmest on record, coinciding globally with the increase in *C. sowerbii* blooms in temperate lakes (Minchin et al., 2016; Marchessaux et al., 2021; Figure 1g). Global climate change and eutrophication trends can dramatically alter the biophysical attributes of rivers and lakes. In particular, the decrease in water transparency due to eutrophication may constitute an ‘invasion window’ for the geographic expansion of *C. sowerbii* (Caputo et al., 2018). Hundreds of reports worldwide have been made in recent decades, and most of them were on jellyfish colonising and flourishing in subtropical and temperate meso‐eutrophic limnic ecosystems where coloured water allows for photoprotection to ultraviolet radiation (Caputo et al., 2018; Fuentes et al., 2019).

The availability of food, together with water quality and temperature, are proposed as key environmental parameters determining FWJ global distribution (Folino‐Rorem et al., 2016). It can be hypothesised that the increased recurrence of jellyfish blooms in lakes is due to long‐term elevated water temperature in their bentho‐pelagic habitats (Marchessaux et al., 2022). While experimental evidence of temperature effects on physiological and behavioural traits exists, other parameters (e.g., pH, dissolved oxygen concentration, the organic load of the water) have only sparsely been studied and predominantly for medusae (reviewed by Acker & Muscat, 1976; Figure 8).

*Craspedacusta sowerbii* medusae commonly act as top predators within respective food webs in newly colonised habitats, feeding predominantly on planktonic grazers (e.g., Dodson & Cooper, 1983), which can reduce phytoplankton biomass and thus control lower trophic levels. Consequently, it was hypothesised that jellyfish blooms transmit top‐down effects indirectly favouring phytoplankton biomass production via trophic cascades, increasing the turbidity of lakes and changing their trophic status (Jankowski et al., 2005). This coincides with recent experimental evidence showing that jellyfish can change vertical nutrient fluxes, i.e., phosphorus and carbon (Schachtl et al., 2023). In contrast to several studies on medusa and polyp feeding (for *Craspedacusta*, but not for *Astrohydra* and *Limnocnida*), rate studies on respiration, excretion, sexual reproduction potential, and mortality are almost completely lacking (e.g., Himchik et al., 2021; Wang et al., 2006), making calculations of daily ration, predation impact, effects of fisheries, and other bioenergetic‐based estimates impossible.

Associated with the predation behaviour of *C. sowerbii* medusae is the evidence that they kill many more prey items than actually consumed (Dodson & Cooper, 1983). Thus, it is reasonable to assume that jellyfish blooms (Figure 1g,h), in addition to facilitating an increase in phytoplankton biomass by depressing herbivores (and sinking unconsumed phytoplankton), promote the sinking of large numbers of zooplankton carcasses that will be mineralised in the hypolimnion of stratified lakes. This, in turn, may promote changes in biogeochemical cycles and near‐bottom hypoxia in hypolimnetic layers during stratification periods, endangering local biodiversity and water quality, and enhancing greenhouse gas emissions. Positive feedback loops in these processes may be expected. However, this is currently investigated neither experimentally nor through modelling. It remains to be seen whether these processes have effects at the ecosystem scale. So‐called ‘jelly falls’, i.e., mass sinking and deposition of gelatinous organism carcasses terminating a bloom (Lebrato et al., 2012), which have been studied in the marine realm to some extent, will contribute at the bloom end to the vertical nutrient flux, but are at present not quantified. Studies in marine environments show that changes in the microbial community can be caused by mass deposition of jellyfish carcasses (Tinta et al., 2012). Comparable studies in freshwater currently do not exist. Also, predator–prey relationships with FWJ medusae (and polyps) as prey are understudied (Dexter et al., 1949; Dodson & Cooper, 1983; Lüskow et al., 2021). A stable isotope study highlighted one key aspect of the invasion success of *C. sowerbii*: While medusae overlapped significantly with other zooplanktivorous predators implying food competition, polyps consumed prey items on the same trophic levels as *Hydra* polyps, but from different communities (Gießler et al., 2023). However, comparable analyses are still missing for other FWJ genera, and no food web model has yet included either of the FWJ life cycle stages.

## 8 COMMUNITY SCIENCE AND PUBLIC INVOLVEMENT

Engaging the public in scientific research, or community science (social media data mining, institutionalised reporting systems, polls), has become an attractive tool for amassing large amounts of cost‐effective data, spanning extensive spatial and temporal scales (Metcalfe et al., 2022), yet its usage for FWJ research remains limited (Lüskow et al., 2021). This probably stems from the well‐documented bias of community scientists towards terrestrial ecosystems and vertebrate taxa (Theobald et al., 2015). Although the marine realm remains the most under‐sampled in that regard (Theobald et al., 2015), probably because of its limited accessibility, oceanic jellyfish appear to attract exceptional attention of community scientists worldwide (Gueroun et al., 2022; Marambio et al., 2021). Whether documenting stranded jellyfish, or photographing them while diving, community scientists offer invaluable cues to current state knowledge (e.g., Baumann & Schernewski, 2012; Doyle et al., 2007) and long‐term trends (e.g., Bernard et al., 2011) of distributions of marine gelatinous fauna, or even to their ecology (e.g., Purcell et al., 2015). The obvious discrepancy between marine and freshwater jellyfish reporting could be attributed to the minute size of FWJ (and benthic life cycle stages), the apparent absence of direct health risks to humans, their seasonal short‐lived blooms, as well as the lack of smartphone apps intended for documenting the FWJ distribution and still poorly advertised governmental/institutional reporting websites. As long as FWJ do not interfere with human activities (e.g., recreating, fishing), they may be of little interest/concern, also for community scientists.

Fortunately, the current development in the field of environmental DNA (eDNA), including fieldable eDNA sequencing (Lewis Ames et al., 2021), offers to fill this gap in FWJ research. This method has already successfully been applied to locating one invasive FWJ species–*C. sowerbii* (Blackman et al., 2022; Jeunen et al., 2022; Mychek‐Londer et al., 2020). No such example exists for any of the other species. There is an emerging notion that community scientists, with very limited training, can produce suitable‐quality samples for eDNA barcoding (Biggs et al., 2015) and it is expected that in the near future, community science and eDNA work will gain importance – also for FWJ research.

## 9 GLOBAL PHYLOGEOGRAPHY OF FRESHWATER JELLYFISH

The inconspicuous nature of the FWJ has hindered their discovery in the past, but is also impairing species delimitation, and as such, affects reasoning on their biogeographic origin and present‐day distribution. The only valid species of *Astrohydra*, *A. japonica* Hashimoto, 1981, was originally described in a freshwater pond in Shizuoka Prefecture and was never documented outside of Japan (Hashimoto, 1981; Peterson et al., 2022). However, the polyp stage of *A. japonica* is indistinguishable from that of another hydrozoan, *Calpasoma dactylopterum* Fuhrmann, 1939, found so far in Europe, North and South America, as well as Israel (Bouillon et al., 2004; Fuhrmann, 1939; Matthews, 1966). The sole difference between the two is that medusa production was not documented in the latter. Whether this is a truly distinguishing feature or merely an effect of culture setup remains unresolved, but for now clearly impacts the understanding of *Astrohydra* phylogeography. As Lewis et al. (2012) stated, without a comparative molecular investigation, a discussion on the possible conspecificity of *Calpasoma* is only speculative.

The FWJ genus *Limnocnida* is considered native to central Africa and India (Jankowski et al., 2008), but some records exist of its presence even in Arizona, USA (Jankowski, 2021). However, the taxonomic ambiguity in species descriptions and the lack of molecular data cast some doubt on the actual number of species contained in this genus (up to six, Jankowski et al., 2008). Some authors even suggested that all *Limnocnida* species might constitute a single, globally distributed species (Bouillon & Boero, 2000). Alternative scenarios imply that the *Limnocnida* ancestor once invaded freshwater habitats from the Tethys Sea (Dumont, 1976; Rao, 1932), which subsequently led to the establishment of a pair of species, with one being native to Africa (*L. tanganjicae*) and another one in India (*L. indica* Annandale, 1912) (Fantham & Porter, 1933; Firoz Ahmad et al., 1987).

The genus *Craspedacusta* presents a similar case; although originally described in the south of England (Lankester, 1880a, 1880b), the genus is thought to be native to the Yangtze Valley (Kramp, 1961) and to have later dispersed globally except Antarctica (Dumont, 1994). Of eleven species formally described, only three to four are considered valid, primarily *C. sowerbii*, *C. kiatingi* Gaw & Kung, 1939, and *C. sinensis* Gaw & Kung, 1939 (Jankowski et al., 2008; Jankowski & Anokhin, 2019; Morpurgo et al., 2021) highlighting the vagueness of the morphological characters utilised to distinguish *Craspedacusta* species. Insufficient morphological documentation of *Craspedacusta* records often precludes their association with a particular species (Oualid et al., 2019). Keeping in mind that sequences from the proximities of the currently disappeared type locality of *C. sowerbii* are not available, taxonomically resolved records based on molecular data suggest that at least two (ITS datasets) and three (Cox1 datasets) *Craspedacusta* lineages should be considered invasive, two of them having spread globally (e.g., Marchessaux et al., 2021; Oualid et al., 2019; Schifani et al., 2019).

When molecular data are analysed in detail, even a more complex, marker‐dependent picture emerges (Lüskow et al., 2021, 2023; Oualid et al., 2019), with numerous examples of multiple invasion events of neighbouring areas, resulting in the presence of distinct haplotypes even at small geographic scales (Fuentes et al., 2019; Lüskow et al., 2023; Morpurgo et al., 2021; Peterson et al., 2022). The general paucity of molecular data and the mitonuclear discordance (e.g., Oualid et al., 2019) necessitate international, collaborative efforts to disentangle the global phylogeography of FWJ with genomic methods, targeting primarily their type localities.

Currently, the majority of FWJ distribution data come from serendipitous observations of their pelagic life cycle stage (Lewis et al., 2012), which is short‐lived and highly seasonal (Marchessaux et al., 2021; Minchin et al., 2016; Rao, 1931). Considering that medusa release is temperature‐dependent (Folino‐Rorem et al., 2016; Marchessaux & Bejean, 2020), and thus some populations may consist of polyps or resting stages (e.g., frustules, podocysts) only, the true geographic ranges of FWJ may be much broader than currently realised. Paraphrasing the title of the work of Duggan and Eastwood (2012), ‘observations of medusae are not enough’, detection of FWJ polyps, however, remains extremely challenging due to their minute body sizes (Acker & Muscat, 1976), which can probably even allow them to avoid detection from more sophisticated methods like eDNA metabarcoding.

The complex life cycle of the FWJ and their wide abiotic tolerance (Lüskow et al., 2021) are likely primary contributors to their immense expansion potential (Jankowski et al., 2008; Peterson et al., 2022), though their exact dispersal trajectories remain speculative. Relocation of minute polyps, resting stages (e.g., Rao, 1931) or motile frustules (Payne, 1924) likely underlies the global distribution of FWJ, particularly of *Craspedacusta* species, as the majority of non‐indigenous populations comprise animals of a single sex (DeVries, 1992; Jarms, 1978; Lüskow et al., 2021). Although not explicitly tested, the capacity to exploit numerous dispersal vectors has often been attributed to the FWJ. Drought‐resistant podocysts are likely involved in aerial dispersion (Dumont, 1994), which appears to be corroborated by the pattern of primary migratory routes of birds (Marchessaux et al., 2021). It is also hypothesised that it was the global aquarium trade (both of plants and animals), or less likely human transportation (particularly during wartime–see comments in Marchessaux et al., 2021) that facilitated their primary dispersal (Lewis et al., 2012; Peterson et al., 2022). Additionally, natural water movements or flooding events may have accounted for a portion of the FWJ distribution (Lewis et al., 2012). Irrespective of means of transportation, the ability to persist and expand in a novel ecosystem is mediated by the innate environmental tolerance ranges of species. There exists evidence that closely related species may be restricted to distinct habitats, like lentic or lotic in the case of *Limnocnida* (Firoz Ahmad et al., 1987); that they may exhibit a particular preference towards artificial or natural waters (Dumont, 1994; Marchessaux et al., 2021), or even tend to distribute at certain altitudes (Fantham & Porter, 1933). With global warming, FWJ will likely be able to bud medusae earlier in the season and allow continuous medusa production (Marchessaux et al., 2022).

## 10 CONCLUDING REMARKS

Approximately 150 years of freshwater jellyfish (FWJ) research have resulted in a substantial amount of information and publications. However, most biologists and environmentalists still have the perception of a ‘new topic’, and indeed, due to global change, FWJ recently gained importance. Historically, our understanding of this small but widespread faunal group was hampered by nomenclatural/taxonomic inconsistencies, underappreciation of some life cycle stages, a predominance of research on one genus, large diversity of publication languages and journals, as well as impacts of colonialism, economic crisis, and global wars. There are parallels to research on marine jellyfish, e.g., the scyphozoan genus *Aurelia* is disproportionally studied because of its widespread occurrence, boom and bust population dynamics, and suitability to cultivate in the laboratory. While in the past, most literature pieces simply reported medusa presence and the conditions at the time of sampling, in recent years, the number of analytical, experimental, and modelling studies has increased considerably. As encouraging as this development is, clearly more research is needed to provide the full picture of FWJ biology and ecology (Figure 8). For example, the relatively well‐documented ecological models of freshwater systems do not incorporate FWJ, except for Gießler et al. (2023). With the observable increase in FWJ occurrences globally, their impacts on the ecology and nutrient flows in aquatic systems are expected to grow, highlighting the need to revise those models. This requires basic information from a variety of sampling methods as well as from original experimental studies on biologically relevant questions. We encourage scientists, not only from biological disciplines, but all, and especially marine jellyfish researchers, to become involved in the ‘old but apparently new’ field of FWJ research that has experienced a never‐seen boom in the first two decades of the 21st century.

## AUTHOR CONTRIBUTIONS

**Florian Lüskow:** Conceptualization (lead); data curation (lead); formal analysis (lead); investigation (lead); methodology (lead); project administration (lead); resources (lead); supervision (lead); validation (lead); visualization (lead); writing – original draft (lead); writing – review and editing (lead). **Nicholas Bezio:** Visualization (supporting); writing – review and editing (supporting). **Luciano Caputo:** Resources (supporting); writing – original draft (supporting); writing – review and editing (supporting). **Xupeng Chi:** Resources (supporting); writing – original draft (supporting); writing – review and editing (supporting). **Henri J. Dumont:** Resources (supporting); writing – review and editing (supporting). **Krishan D. Karunarathne:** Resources (supporting); writing – review and editing (supporting). **Pablo J. López‐González:** Resources (supporting); writing – review and editing (supporting). **Maciej K. Mańko:** Resources (supporting); writing – original draft (supporting); writing – review and editing (supporting). **Guillaume Marchessaux:** Resources (supporting); visualization (supporting); writing – original draft (supporting); writing – review and editing (supporting). **Kentaro S. Suzuki:** Resources (supporting); writing – original draft (supporting); writing – review and editing (supporting). **Evgeny A. Pakhomov:** Conceptualization (supporting); funding acquisition (lead); resources (supporting); writing – review and editing (supporting).

## CONFLICT OF INTEREST STATEMENT

The authors declare that there is no conflict of interest.

## Supporting information


Figures S1–S3.

## Data Availability

The literature database has been deposited in PANGAEA (https://doi.org/10.1594/PANGAEA.962186).
