# Supplementary material for: Hidden gems: Scattered knowledge hampered freshwater jellyfish research over the past one‐and‐a‐half centuries
Source: Ecol Evol. 2024 Sep 29;14(10):e70350. doi: 10.1002/ece3.70350 (PMC11439511; doi:10.1002/ece3.70350)
Supplement: Supplementary file 1 — Figures S1–S3. [file ECE3-14-e70350-s001.docx]

Supplementary Materials

Hidden gems: Scattered knowledge hampered freshwater jellyfish research over the past one-and-a-half centuries

Florian Lüskow^1,2*^, Nicholas Bezio^3^, Luciano Caputo^4,5^, Xupeng Chi^6,7^, Henri J. Dumont^8,9^, Krishan D. Karunarathne^10^, Pablo J. López-González^11^, Maciej K. Mańko^12^, Guillaume Marchessaux^13^, Kentaro S. Suzuki^14^, Evgeny A. Pakhomov^1,2^

^1^ Department of Earth, Ocean and Atmospheric Sciences, University of British Columbia, Vancouver, Canada

^2^ Institute for the Oceans and Fisheries, University of British Columbia, Vancouver, Canada

^3^ Department of Biology, University of Maryland, Baltimore, USA

^4^ Facultad de Ciencias, Instituto de Ciencias Marinas y Limnolóogicas, Universidad Austral de Chile, Valdivia, Chile

^5^ Centro Transdisciplinario de Estudios Ambientales y Desarrollo Humano Sostenible, Universidad

Austral de Chile, Valdivia, Chile

^6^ CAS Key Laboratory of Marine Ecology and Environmental Sciences, Institute of Oceanology, Chinese Academy of Sciences, Qingdao, China

^7^ Laboratory of Marine Ecology and Environmental Sciences, Qingdao National Laboratory for Marine Science and Technology, Qingdao, China

^8^ Department of Biology, Gent University, Ghent, Belgium

^9^ Department of Ecology, Jinan University, Guangzhou, China

^10^ Department of Aquaculture and Fisheries, Wayamba University of Sri Lanka, Makandura, Gonawila (NWP), Sri Lanka

^11^ Biodiversidad y Ecología Acuática, Departamento de Zoología, Universidad de Sevilla, Sevilla, Spain

^12^ Laboratory of Plankton Biology, Department of Marine Biology and Biotechnology, University of Gdańsk, Gdynia, Poland

^13^ Laboratory of Ecology, Department of Earth and Marine Science, University of Palermo, Palermo, Italy

^14^ Sustainable System Research Laboratory, Central Research Institute of Electric Power Industry, Abiko, Japan


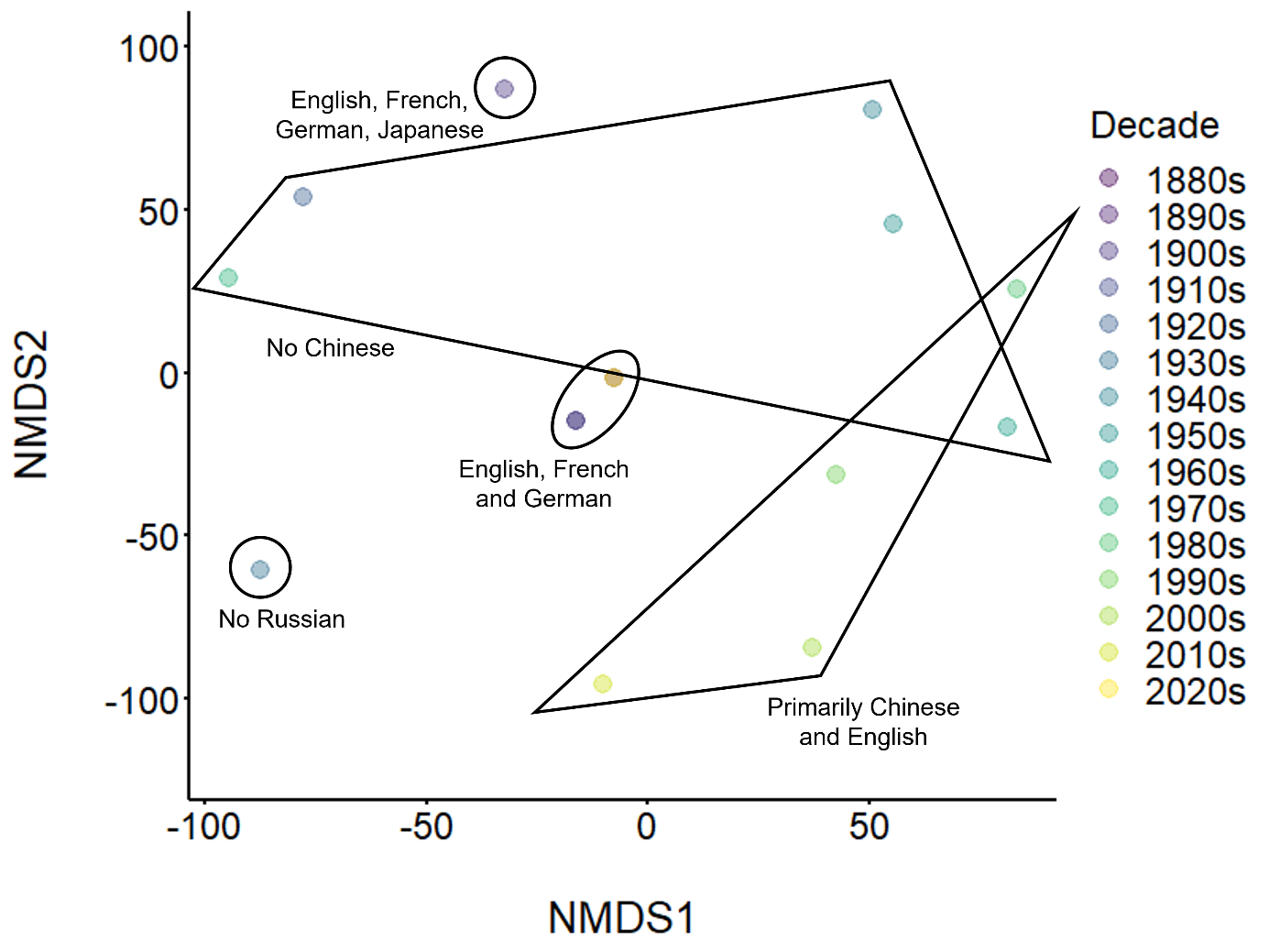


**Figure S1.** Non-metric multidimensional scaling ordination of primary papers on freshwater jellyfish worldwide grouped by decade (1880s–2020s) according to their publication language (English, German, French, Chinese, etc.). Similarity between language data was calculated by means of Bray-Curtis measurements based on family densities (stress level = 0.213).


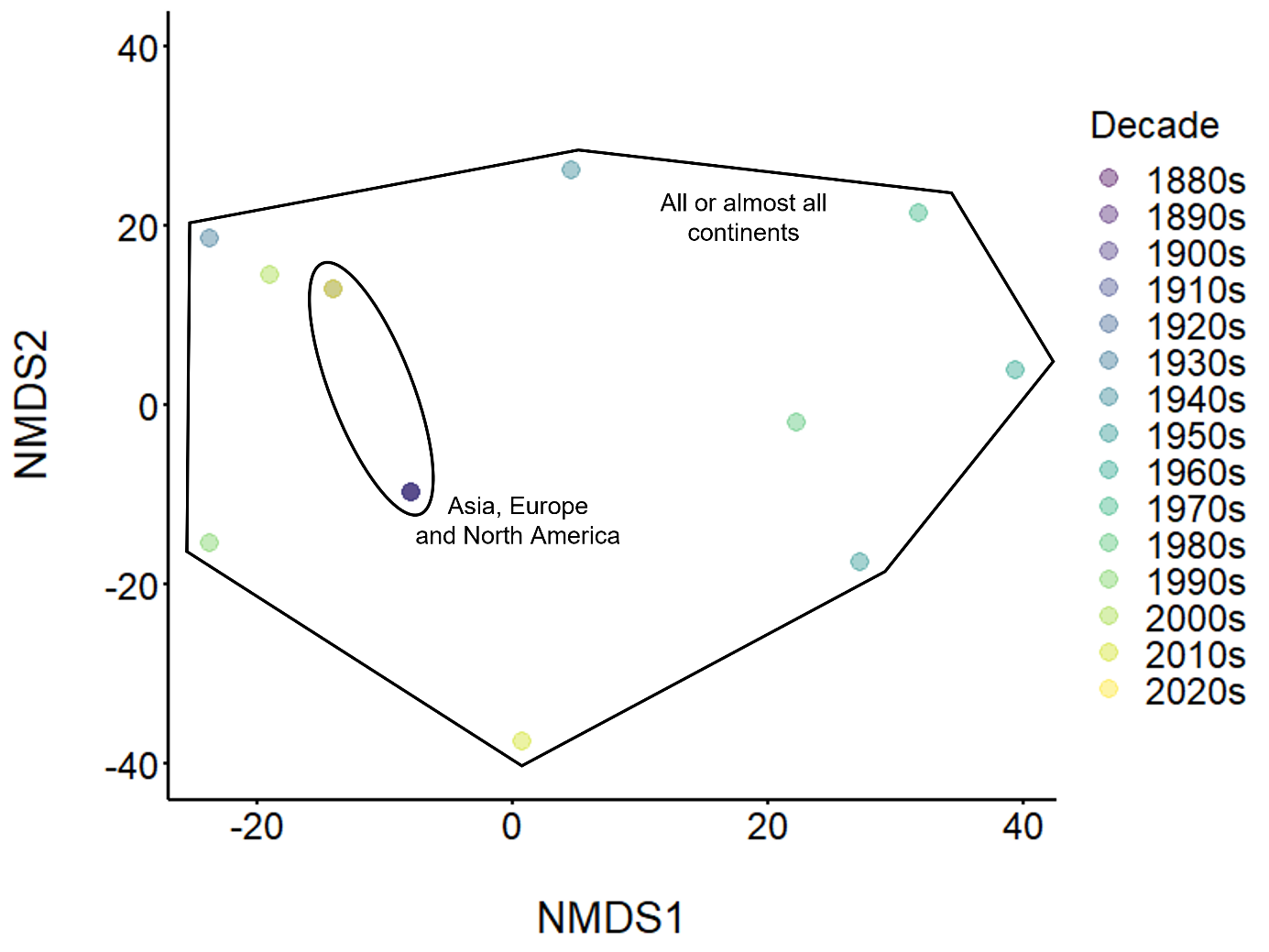


**Figure S2.** Non-metric multidimensional scaling ordination of primary papers on freshwater jellyfish worldwide grouped by decade (1880s–2020s) according to the continent of the first author's primary affiliation (Africa, Asia, Europe, North America, Oceania, and South America). Similarity between continent data was calculated by means of Bray-Curtis measurements based on family densities (stress level = 0.211).


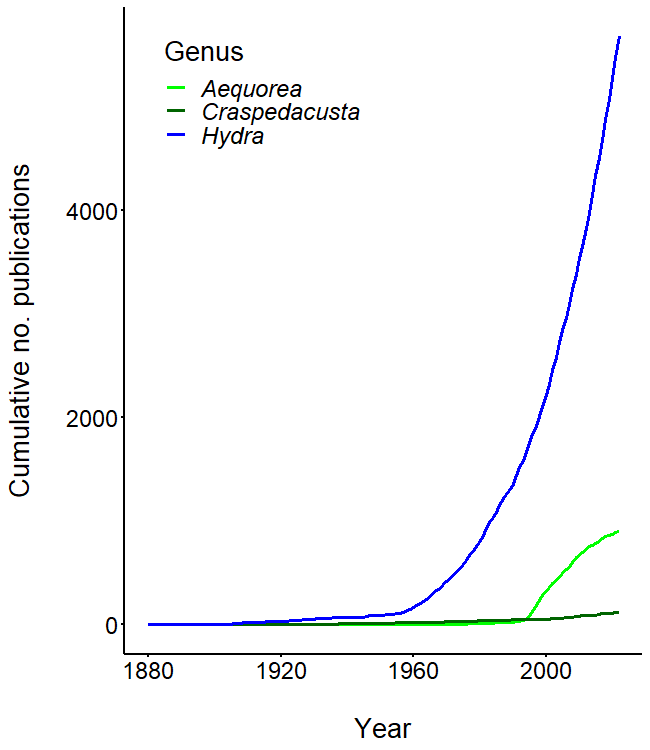


**Figure S3.** The cumulative number of primary papers on the genera *Aequorea* (well-studied marine hydrozoan, N = 903), *Craspedacusta* (best-studied freshwater jellyfish, N = 124), and *Hydra* (best-studied freshwater-hydrozoan, N = 5673) worldwide between 1880 and 2022. Data were extracted from the Web of Science (WoS) using 'Year Published' and the genus name as 'Topic' on 2 May 2023.
